# Endobronchial Valves for the Treatment of Bronchopleural Fistula and Pneumothorax Caused by Pulmonary Cryptococcosis in an AIDS Patient

**DOI:** 10.3389/fmed.2020.00051

**Published:** 2020-02-18

**Authors:** Ying Wen, Chao-nan Liang, Ying Zhou, Hai-feng Ma, Gang Hou

**Affiliations:** ^1^Department of Infectious Diseases, First Hospital of China Medical University, Shenyang, China; ^2^Department of Pulmonary and Critical Care Medicine, First Hospital of China Medical University, Shenyang, China

**Keywords:** endobronchial valves, bronchopleural fistula, pneumothorax, cryptococcosis, AIDS

## Abstract

Cryptococcal disease is an opportunistic infection that occurs primarily among people with advanced HIV disease and is an important cause of morbidity and mortality. Spontaneous pneumothorax (SP) is rare in acquired immune deficiency syndrome (AIDS) patients with pulmonary cryptococcosis (PC), but when it occurs, rapid and effective treatment is crucial to the prognosis, with mortality rates varying from 30 to 60%. SP is related to pneumonia mainly due to bacterial infections and pneumocystic *jirovecii* pneumonia (PJP). However, SP caused by PC is rare. When it occurs, it is often fatal and refractory, which is a challenge both for patients and clinicians. Here, we report a case of SP during the treatment of cryptococcal disease in a patient with AIDS. The pneumothorax remained despite chest tube drainage and evolved into a bronchopleural fistula that was confirmed by the Chartis system. The pneumothorax was significantly resolved following the placement of 2 endobronchial valves (EBVs). The patient tolerated the procedure very well and the pneumothorax gradually resolved. When immunocompromised patients suffer from refractory pneumothorax or prolonged air leaks, EBV implantation may be a feasible and minimally invasive procedure for this vulnerable population.

## Background

Cryptococcal disease is an opportunistic infection that occurs primarily among people with advanced human immunodeficiency virus (HIV) and is an important cause of morbidity and mortality (1, 2). Spontaneous pneumothorax (SP) is a well-recognized complication of acquired immune deficiency syndrome (AIDS) and is also a major factor that affects patient prognosis. AIDS-related SP is often recurrent and refractory to chest tube drainage. Interventional bronchoscopy can help rapidly confirm the presence of a fistula and administer precise treatment. Here, we report a case of AIDS-related SP in a patient infected with cryptococcus, resulting in a bronchopleural fistula that was successfully treated by the placement of endobronchial valves (EBVs) through a bronchoscope.

## Case Presentation

**Figure 1:**
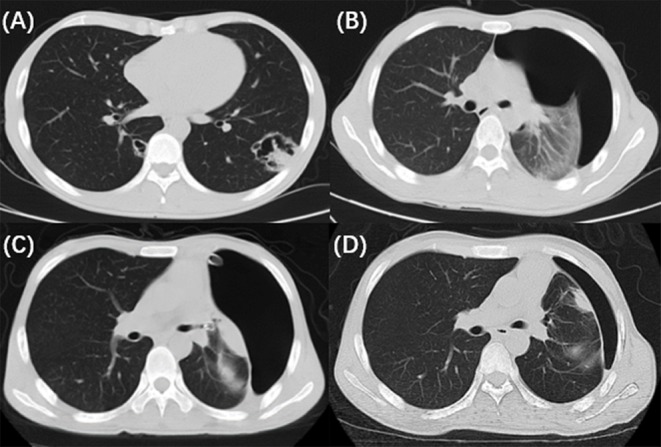
The radiological and bronchoscopic imaging manifestation. **(A)** Chest CT revealed an irregular thick-walled cavitation in the left lower lobe and cystic lesions in the right lower lobe. **(B)** Repeat chest CT on the 18th day showed pneumothorax in the left lung. **(C)** A follow-up chest CT (2 days after EBV implantation) showed partial regression of the pneumothorax. **(D)** Chest CT acquired after the valves removed revealed that the pneumothorax improved gradually.

A 25-year-old man was admitted to our hospital for intermittent fever, non-productive cough, headache and vomiting for 2 weeks. On admission, his vital signs were as follows: temperature, 36.5°C; respiratory rate, 16 bpm; pulse, 80 bpm; and blood pressure, 102/57 mmHg. the laboratory data revealed a white blood cell (WBC) count of 4.29 × 109/L (3.50–9.50^*^109/L), a neutrophil ratio (NE%) of 84.4% (40.0–75.0%), a C-reactive protein (CRP) level of 71.7 mg/L (0–5.00 mg/L) and a procalcitonin (PCT) level of 0.06 ng/mL (0–0.05 ng/mL). The HIV antibody confirmation test was positive, with a ${\text{CD}}_{4}^{+}$ T-cell count of 25 cells/μL. The cerebrospinal fluid (CSF) smear and blood culture were positive for Cryptococcus neoformans. The other routine biochemistry results were normal. Chest computed tomography (CT) revealed an irregular thick-walled cavitation in the left lower lobe and cystic lesions in the right lower lobe (Figure 1A). The diagnosis was AIDS with cryptococcal meningitis and pulmonary cryptococcosis (PC). Antifungal treatment was started with intravenous amphotericin B (1 mg/ kg/day) and oral flucytosine (100 mg/kg/day).

**Figure 2:**
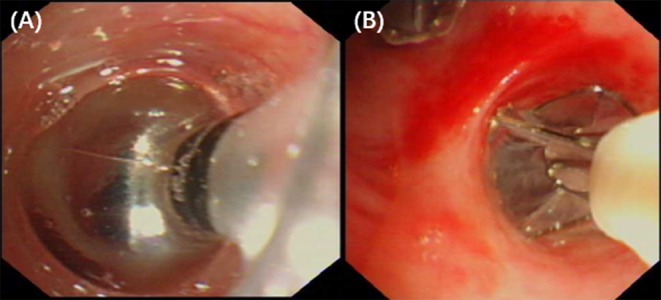
**(A)** Bronchoscopy revealed that the balloon of the Chartis system occluded the lobar bronchus to identify the lung segments with air leaks. **(B)** Bronchoscopy revealed two EBVs were implanted into the airway of LB3 and LB4+5.

**Figure 3:**
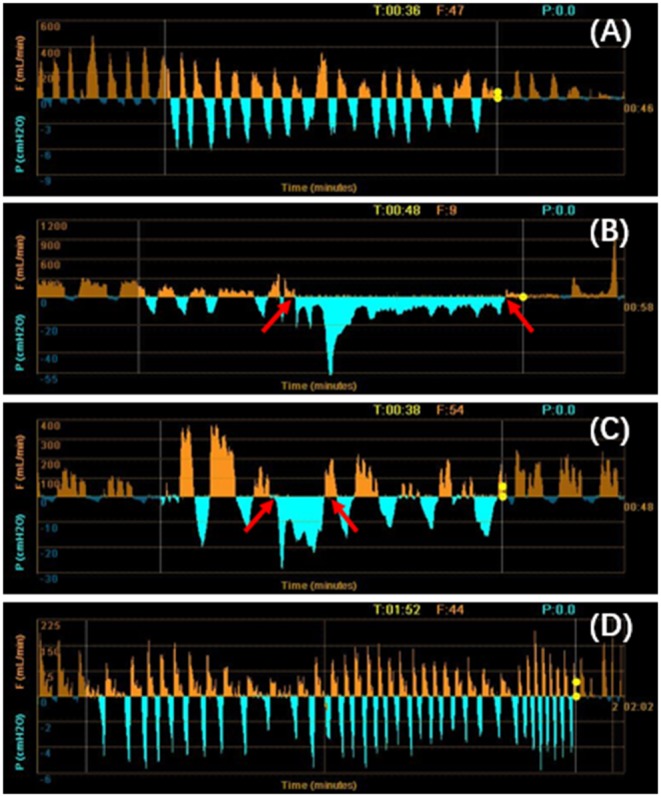
**(A–D)** Using the Chartis system to identify the lung segments with air leaks. When the balloon occluded the LB4+5 **(B)** and LB3 **(C)**, a negative pressure was exerted to the exhaust port of the thoracic drainage bottle. The patient's inspiratory pressure was overlapped with the negative pressure created by the vacuum. The negative pressure generated by the vacuum was displayed as a low-level continuous negative pressure at both the inspiratory and expiratory phases (between arrows). It was the sign of the presence of air leaks. There were no air leaks in the left lower lobe **(A)** and LB1+2 **(D)**. T, time; P, pressure; F, flow.

On the 18th day of hospitalization, the patient suddenly felt severe chest pain and dyspnea. A repeated chest CT showed pneumothorax (Figure 1B). A chest tube was placed on his left side and yielded symptomatic improvement. Four weeks later, a follow-up CT showed that his left lung did not re-expand. The diagnosis of persistent air leaks (PALs) was confirmed. The patient presented intermittent spasms secondary to cryptococcal meningitis and respiratory failure [Partial pressure of oxygen/Fractional inspired oxygen concentration (PaO_2_/FiO_2_): 227.4, FiO_2_: 33%]. Because the patient was weak and in poor clinical condition (respiratory rate, 25 bpm; pulse, 106 bpm; and blood pressure, 95/58 mmHg.), had respiratory failure and intermittent spasms, and was not a surgical candidate, we inserted EBVs to block the air leak and facilitate healing. The source of the air leaks was in LB3 and LB4+5, which was identified by the Chartis system (Pulmonx SARL, CHARTIS CONSOLE, Switzerland). The Chartis system consists of a balloon catheter and a console that houses flow and pressure sensors (Figure 2A). Airflow is measured through the sensors in the console. When assessing an airway that is exposed to a leak, due to the strong negative pressure in the pleural cavity, the Chartis system visually displays an abnormal block of constant negative pressure (Figure 3). Two EBVs (EBV-TS-4.0 and EBV-TS-5.5) were implanted into the desired airway (Figure 2B).

The patient tolerated the procedure very well with an immediate reduction in air leakage and subjective alleviation of dyspnea. A follow-up chest CT 1 week later showed partial regression of the pneumothorax (Figure 1C). Two months later, the valves were removed. The pneumothorax improved gradually (Figure 1D), and no adverse events related to the EBVs were observed.

## Discussion/Conclusion

Spontaneous pneumothorax is a rare but serious complication of opportunistic infections in AIDS patients, with mortality rates varying from 30 to 60% (3), and is related to pneumonia mainly due to bacterial infections and pneumocystic *jirovecii* pneumonia (PJP) (4). However, SP caused by PC is rare. When it occurs, it is often fatal and refractory, which is a challenge both for patients and clinicians.

Generally, chest tube evacuation of intrathoracic air using a water seal or surgery with a thoracoscopic poudrage/pleurectomy remains the standard treatment for SP (5). Interventional bronchoscopy is helpful for the treatment of SP. The EBV system (Zephyr EBV), works as a one-way valve and is placed in the targeted airway proximal to the area of the air leak, preventing the entrance of air during inspiration and for the drainage of bronchial secretions, thereby enabling the lung to re-expand and heal (6). Early bronchoscopic intervention in patients with AIDS-related SP may shorten the duration of chest tube drainage and help improve the disease prognosis.

There are other minimally invasive and bronchoscopic therapeutic options for prolonged air leakage, such as endobronchial Watanabe spigot (EWS), a type of silicone bronchial blocker. Previous studies revealed that EWS insertion was an effective modality for managing prolonged air leaks caused secondary to pneumothorax, empyema, and postoperative complications (7–9). Infection control at the target bronchus is important for a prolonged duration of EWS indwelling. EWSs should be placed after achieving infection control with antibiotic therapy (10). Chest tube and underwater seal drainage should also be placed to drain the empyema and gain control of the infection.

In contrast to other blocking devices, EBVs allow for expiration and the clearance of bronchial secretions, thereby reducing the risk of post obstructive pneumonia (11). Furthermore, EBVs can be easily removed when the air leaks are resolved to allow adequate time for tissue healing (11). We summarized the published cases using EBVs for PAL(s) in Table 1 (12–27). Only three cases were of patients with AIDS, and our case was the only patient whose SP was secondary to PC. EBVs are well-tolerated and effective for the treatment of PALs. Complications are rare and include pneumonia (13), migration of valves (17), and bacterial colonization. It is important to identify the source of the air leak. We can use a chest drainage system to assess the air leaks. A balloon occlusion test was performed to identify the affected region. The balloon was inflated to achieve complete occlusion in the lobar, segmental, and subsegmental bronchi. The affected airway was identified by the reduction or elimination of the air leak through the chest tube 15–20 s after the occlusion. The Chartis system (Pulmonx) is also a good choice for detecting air leaks, as previously mentioned (28, 29). When the source of the leakage is confirmed, the Chartis system is helpful for confirming the absence of collateral ventilation in the target bronchus which is a key factor for a favorable clinical response. Collateral ventilation from the adjacent lobes through collateral channels could prevent target lobe atelectasis, which potentially limits the clinical response after endoscopic bronchial occlusion (29).

**Table 1:** Summary of patients' demographics and characteristics for the published cases using EBV for PAL(s).

**Patients**	**Age/Gender**	**Reason for PAL(s)**	**Underlying lung disease**	**Lobe(s) treated**	**Valves placed(n)**	**Time of air leak stopped**	**Complications**
1	35/F	Lung transplant	Lymphangioleiomyomatosis	LUL, lingula	4	Null	/
2	32/F	Lung abscess	CAP, ARDS	RUL	3	2 h	Infection in the residual airspace
3	63/F	Post-ablation tumor necrosis	Left lobectomy due to NSCLC	LUL, LLL	2	15 min	/
4	62/M	Pulmonary resection	Lung ca, COPD, emphysema	LUL	1	2 days	No
5	28/F	ECMO	Fontan Syndrome	LUL	1	Immediately	No
6	60/M	Bullectomy	Emphysema	LUL, RUL	3	/	No
7	57/F	Lobectomy	Lung adenocarcinoma	LUL	1	2 days	No
8	69/M	Emphysema rupture	COPD	LLL	1	1 week	No
9	61/M	Giant bullectomy	Bullous emphysema	LUL	2	3 days	Recurrent chest infections
10	63/M	Microwave ablation	SCC	RUL, RLL	3	Null	Cough
11	58/F	Microwave ablation	Suspected NSCLC	RUL	1	Several days	No
12	71/F	Microwave ablation	Adenocarcinoma	LLL	1	15 days	No
13	68/M	Empyema	Lobectomy due to pulmonary adenocarcinoma	RLL	4	/	No
14	61/M	Thoracotomy	SCC, pneumoconiosis	RUL	3	3 days	No
15	56/M	Deflated giant bulla	Bullous emphysema	RUL	3	2 days	No
16	60/M	Placement of drainage in GEB	GEB	LUL	2	1 day	No
17	67/M	Pleurectomy	Empyema	LUL	1	1 day	No
18	39/M	Pleurectomy	Empyema		2	5 days	No
19	75/M	SP	Emphysema	LUL	1	1 day	No
20	21/M	PJP	AIDS	RUL	3	Several days	No
21	49/M	SP	COPD	LUL	1	2 days	No
22	32/M	Loculated empyema	Null	RML	1	5 days	No
23	43/F	Chest tube drainage	Fungal empyema	RML	2	3 days	/
24	38/F	Mechanical ventilation	Organizing pneumonia	RUL, RML	7	13 days	No
25	60/M	Mechanical ventilation	Influenza A pneumonia	LUL	2	14 days	No
26	42/M	PJP	AIDS	RUL	3	8 days	No
27	60/M	Emphysema rupture	COPD	RUL	3	1 day	No

*F, Female; M, Male; LUL, Left upper lobe; CAP, Community acquired pneumonia; ARDS, Adult respiratory distress syndrome; RUL, Right upper lobe; NSCLC, Non-small cell lung cancer; LLL, Left lower lobe; Lung ca, Lung cancer; COPD, Chronic obstructive pulmonary disease; ECMO, Extra-corporeal membrane oxygenation; RLL, Right lower lobe; SCC, Squamous-cell carcinoma; GEB, Giant emphysematous bulla; SP, Spontaneous pneumothorax; PJP, P. jirovecii pneumonia; AIDS, Acquired immune deficiency syndrome; RML, Right middle lobe*.

In conclusion, SP caused by PC is rare but life threatening, and EBV placement may be a good choice for patients with AIDS who are non-surgical candidates due to their extremely poor condition.

## Data Availability Statement

All datasets for this study are included in the article.

## Ethics Statement

Written informed consent was obtained from the participant for the publication of this case report and any potentially identifying images/information.

## Author Contributions

GH made substantial contributions to the conception and design of the work. GH, YW, and YZ helped to collect the data from the cases. GH, YW, and CL wrote the manuscript. GH and YW interpreted the data for the work. GH and HM performed the bronchoscopy. All authors revised the paper critically for important intellectual content, provided final approval of the version to be published, agreed to be accountable for all aspects of the work in ensuring that questions related to the accuracy or integrity of any part of the work are appropriately investigated, and resolved and contributed toward the acquisition of data for the work.

### Conflict of Interest

The authors declare that the research was conducted in the absence of any commercial or financial relationships that could be construed as a potential conflict of interest.

## Abbreviations

AIDS: Acquired immune deficiency syndrome
SP: Spontaneous pneumothorax
PC: Pulmonary cryptococcosis
EBV: Endobronchial valve
WBC: White blood cell
NE%: Neutrophil ratio
CRP: C-reactive protein
PCT: Procalcitonin
CSF: cerebrospinal fluid
CT: Computed tomography
PALs: Persistent air leaks.
